# Supplementary material for: Assessing Adherence, Competence and Differentiation in a Stepped-Wedge Randomised Clinical Trial of a Complex Behaviour Change Intervention
Source: Nutrients. 2020 Aug 4;12(8):2332. doi: 10.3390/nu12082332 (PMC7469004; doi:10.3390/nu12082332)
Supplement: Supplementary file 1 [file nutrients-12-02332-s001.pdf]

	Wave 1	Wave 2	Wave 3	Wave 4	Wave 5
Hospital 1	Control	Intervention	Intervention	Intervention	Intervention
Hospital 2	Control	Control	Intervention	Intervention	Intervention
Hospital 3	Control	Control	Control	Intervention	Intervention
Hospital 4 and 5[a]	Control	Control	Control	Control	Intervention

[a] Training occurred at the same time as these study sites shared a dietetic department.

Supplementary Figure 1 Sequence of intervention roll-out in the stepped-wedge design.

Supplementary File 2.

Inter- and Intra- Rater Reliability

Inter-rater reliability (between the two coders) and intra-rater reliability (for each coder) for the study specific checklist was assessed using Cohen's kappa. For the BECCI and CTS-R competence and interpersonal effectiveness items, inter-rater and intra-rater reliability were assessed using two-way mixed, absolute agreement, single measure intraclass correlation coefficients (ICC). All indices of inter-rater and intra-rater reliability were interpreted according to published guidelines[1]: 0.01 ('poor' agreement); 0.02-0.2 ('slight' agreement); 0.21 to 0.40 ('fair' agreement); 0.41 to 0.60 ('moderate' agreement); 0.61 to 0.80 ('substantial' agreement); 0.81 to 1 ('almost perfect' agreement).

The level of inter-rater and intra-rater reliability achieved is summarised in Supplementary Table One. The majority of items within and between raters fell within the substantial to almost perfect range. Lower inter-rater reliability (slight to fair) was demonstrated by one study checklist item and both cognitive therapy scale items.

Supplementary Table One.

Summary of inter and intra-rater reliability for behaviour change outcomes

	Study Specific Checklist						BECCI	Cognitive Therapy Scale	
	Eating as Integral to RT	Reasons for RT	Written Nutrition Plan	Written Nutrition Plan Reviewed	Validated Nutrition Assessment	Adequacy of Intake	Overall Practitioner Score	Competence	Interpersonal Effectiveness
Inter-rater Reliability									
Cohen's Kappa	.73 (Substantial)	1.0 (Almost perfect)	.61 (Substantial)	.81 (Almost perfect)	0.84 (Almost perfect)	0.15 (Slight)	---	---	---
ICC Single Measures	---	---	---	---	---	---	0.56 (Moderate)	0.38 (Fair)	0.15 (Slight)
Intra-rater Reliability (Rater One)									
Cohen's Kappa	.95 (Almost perfect)	1.0 (Almost perfect)	1.0 (Almost perfect)	.93 (Almost perfect)	.97 (Almost perfect)	.79 (Substantial)	---	---	---
ICC Single Measures	---	---	---	---	---	---	.94 (Almost perfect)	.91 (Almost perfect)	.95 (Almost perfect)
Intra-rater Reliability (Rater Two)									
Cohen's Kappa	1.0 (Almost perfect)	1.0 (Almost perfect)	.96 (Almost perfect)	.95 (Almost perfect)	.93 (Almost perfect)	1.0 (Almost perfect)	---	---	---
ICC Single Measures	---	---	---	---	---	---	.91 (Almost perfect)	.82 (Almost perfect)	.87 (Almost perfect)

1. Landis JR, Koch GG. The Measurement of Observer Agreement for Categorical Data. *Biometrics.* 1977;33(1):159-174.
